# Comparison of tumor and normal tissue dose for accelerated partial breast irradiation using an electronic brachytherapy eBx source and an Iridium‐192 source

**DOI:** 10.1120/jacmp.v11i4.3301

**Published:** 2010-09-14

**Authors:** Salahuddin Ahmad, Daniel Johnson, Jessica R. Hiatt, D. Timothy Still, Eli E. Furhang, David Marsden, Frank Kearly, Damian A. Bernard, Randall W. Holt

**Affiliations:** ^1^ Department of Radiation Oncology University of Oklahoma Health Sciences Center Oklahoma OK; ^2^ Rhode Island Hospital Providence, RI; ^3^ Wellstar Kennestone Hospital Marietta GA; ^4^ Beth Israel Medical Center New York NY; ^5^ White Plains Hospital Dickstein Cancer Center White Plains NY; ^6^ Maryland Regional Cancer Center Silver Spring MD; ^7^ Rush University Medical Center Chicago IL; ^8^ Enloe Medical Center Chico CA USA

**Keywords:** accelerated partial breast irradiation, electronic brachytherapy, Iridium HDR

## Abstract

The objective of this study has been to compare treatment plans for patients treated with electronic brachytherapy (eBx) using the Axxent System as adjuvant therapy for early stage breast cancer with treatment plans prepared from the same CT image sets using an Ir‐192 source. Patients were implanted with an appropriately sized Axxent balloon applicator based on tumor cavity size and shape. A CT image of the implanted balloon was utilized for developing both eBx and Ir‐192 brachytherapy treatment plans. The prescription dose was 3.4 Gy per fraction for 10 fractions to be delivered to 1 cm beyond the balloon surface. Iridium plans were provided by the sites on 35 of the 44 patients enrolled in the study. The planning target volume coverage was very similar when comparing sources for each patient as well as between patients. There were no statistical differences in mean %V100. The percent of the planning target volume in the high dose region was increased with eBx as compared with Iridium (p<0.001). The mean maximum calculated skin and rib doses did not vary greatly between eBx and Iridium. By contrast, the doses to the ipsilateral lung and the heart were significantly lower with eBx as compared with Iridium (p<0.0001). The total nominal dwell times required for treatment can be predicted by using a combination of the balloon fill volume and planned treatment volume (PTV). This dosimetric comparison of eBx and Iridium sources demonstrates that both forms of balloon‐based brachytherapy provide comparable dose to the planning target volume. Electronic brachytherapy is significantly associated with increased dose at the surface of the balloon and decreased dose outside the PTV, resulting in significantly increased tissue sparing in the heart and ipsilateral lung.

PACS numbers: 87,53.Jw, 87.55.dk, 87.55.D‐,87.56 b‐,87.56.bg

## I. INTRODUCTION

Breast cancer will be diagnosed in an estimated 192,370 women in 2009.^(^ ^1^ ^)^ Approximately 60% of these cases will have cancer confined to the breast. Breast conserving therapy (BCT) for localized breast cancer is widely accepted and involves surgery usually followed by radiation treatment. However, over 40% of women choose a mastectomy over BCT and many who do choose BCT forego the radiation therapy component of their treatment program.^(^ ^2^ ^,^ ^3^ ^)^ Reasons cited often include travel distance to a radiation treatment center and time away from home and work for a six to seven week course of whole breast external beam radiation.^(^ ^4^ ^–^ ^6^ ^)^

Several methods of accelerated partial breast irradiation (APBI) have been developed to irradiate a smaller volume of tissue with a larger dose per fraction. This allows the prescription dose to be delivered over the course of one week with an expected decrease in dose to normal tissue including the heart, lungs and contralateral breast compared with whole breast external beam therapy. Interstitial multi‐catheter brachytherapy has demonstrated that APBI can be effective; however, technical challenges have prevented this form of APBI from being widely adopted.^(^ ^7^ ^)^

Balloon‐based brachytherapy has achieved wider acceptance due to the ease of use. The MammoSite balloon‐based treatment utilizes an Iridium‐192 source to provide APBI, and local control rates at five‐year follow‐up have been good. Benitez et al.^(^ ^8^ ^)^ reported results of an initial intracavitary APBI study and no local recurrences (either at the tumor bed or elsewhere in the breast) or regional recurrences have occurred in the 36 patients who have been followed for a median of 5.5 years. However, the use of radioisotopes requires a shielded radiation vault and a high‐dose rate (HDR) afterloader unit, and many patients are unable or unwilling to travel to a fully‐equipped facility.^(^ ^9^ ^)^

The Axxent balloon‐based treatment uses a miniature X‐ray source operating at 50 kVp, which reduces the shielding requirements and avoids the handling and storage constraints required for radioisotope sources, making widespread use of APBI economically feasible.^(^ ^10^ ^)^ Early results of the first clinical study of the Axxent eBx system were favorable with complication rates similar to Ir‐192 APBI.^(^ ^11^ ^)^

Several studies^(^ ^9^ ^)^ have compared the dosimetric characteristics of these two balloon‐based systems; however, in those studies the eBx treatment plans were simulated from patients actually treated with an Ir‐192 source. We herein report the dosimetric comparison of these two forms of APBI that starts with eBx treatment plans for patients with localized breast cancer.

## II. MATERIALS AND METHODS

Treatment plans for 35 patients were included in these analyses. Patients were enrolled in a study protocol that was approved by an Institutional Review Board at each participating site. The study was conducted in accordance with the Declaration of Helsinki and all applicable regulations. Details of the study methods have been reported elsewhere.^(^ ^11^ ^)^

Patients selected in this study were greater than 50 years old with completely resected T1 invasive ductal cancer or ductal carcinoma *in situ* less than 2 cm in diameter. Most patients were postmenopausal Caucasian women with no family history of cancer. Each patient was implanted with an appropriately‐sized Axxent balloon applicator (Xoft, Inc., Sunnyvale, CA) based on the size and shape of their tumor cavity. After implantation, the balloon was inflated with sterile saline to an optimum size as evaluated by computed tomography (CT) imaging. The CT images of the implanted balloon were used for developing both the eBx and the Ir‐192 treatment plans using BrachyVision (Varian Medical Systems, Palo Alto, CA) treatment planning system (TPS) or Plato TPS (Nucletron, Columbia, MD). The TG‐43U method for modeling brachytherapy dose distribution was used to compute dose distributions for both the eBx source and Ir‐192.^(^ ^12^ ^,^ ^13^ ^)^ The TG‐43U model assumes a uniform water scattering media, and while it may not fully describe the dosimetry, it was chosen for this study as it was the most commonly available commercial model at the time. The prescription dose was 3.4 Gy per fraction for 10 fractions to be delivered to planning target volume (PTV) that covers the volume 1.0 cm beyond the balloon surface.

**Figure 1:**
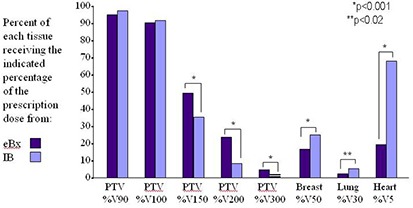
Comparison of the percentage of the planned target volume (PTV), breast, lung and heart receiving a given percentage of the prescription dose based on the eBx and Ir‐192 treatment plans.

Dose‐volume histogram (DVH) metrics were compared between eBx and Ir‐192 treatment plans. The dosimetric data in this report includes the percent of the PTV receiving 90%, 100%, 150%, 200% and 300% of the prescribed dose as shown in Fig. 1; the maximum calculated dose to the skin and rib (Gy); and the percentages of the ipsilateral breast, ipsilateral lung and heart volumes receiving 50%, 30% and 5%, respectively, of the prescription dose for both sets of treatment plans. A t‐test was used to compare mean percents between the eBx group and the Ir‐192 group. The Ir‐192 plans were individually optimized for each patient.

The correlation of total nominal dwell times (TNDT) required for the planned treatments using the eBx method with the balloon fill volume and PTV at implant time was studied. The TNDT is the summation of the dwell times at all planned dwell points.

## III. RESULTS

Comparisons of the DVH metrics show that the PTV coverage was similar between the two treatment modalities (Table 1). There were no statistical differences in mean %V100. The percent of the planning target volume in the high‐dose regions (%V150, %V200 and %V300) was significantly increased with eBx as compared to Ir‐192 (Table 2). While the mean maximum skin dose was 10% less with eBx than with Ir‐192, it was not drastically different. The mean maximum rib doses did not vary greatly between eBx and Ir‐192. By contrast, the doses to the ipsilateral breast (%V50) and ipsilateral lung (%V30), however, were dramatically lower in the eBx treatment plan as compared to those using Ir‐192 (Table 3). In the left sided cases, the heart dose was considerably lowered with the use of eBx.

**Table 1:** Comparison of planning target volume (PTV) coverage.

	*eBx %V90*	*Ir‐192 %V90*	*eBx %V100*	*Ir‐192 %V100*
Mean SD	95.45 3.27	97.28 3.05	89.80 4.46	90.58 5.11
t‐test	p=0.0003		p=0.2759	

**Table 2:** Comparison of dose in the high‐dose regions.

	*eBx %V150*	*Ir‐192 %V150*	*eBx %V200*	*Ir‐192 %V200*	*eBx %V300*	*Ir‐192 %V300*
Mean SD	48.76 7.86	35.10 8.59	22.98 7.23	7.98 6.16	3.50 3.41	0.47 0.64
t‐test	p<0.0001		p<0.0001		p<0.0001	

**Table 3:** Comparison of dose to normal tissues of the skin, rib, breast, lung and heart.

	*Max Skin Dose (Gy/Fraction)*		*Max Rib Dose (Gy/Fraction)*		*Ipsilateral Breast %V50*		*Ipsilateral Lung %V30*		*Heart %V5 Left Side Treatment (n = 23)*	
	*eBx*	*Ir‐192*	*eBx*	*Ir‐192*	*eBx*	*Ir‐192*	*eBx*	*Ir‐192*	*eBx*	*Ir‐192*
Mean SD	2.78 1.54	3.09 1.09	3.65 2.73	3.38 1.75	18.72 6.5	24.85 7.21	1.10 1.12	5.15 9.38	19.52 16.68	68.26 24.32
t‐test	p=0.1142		p=0.1663		p=<0.0001		p=0.0107		p<0.0001	

**Figure 2:**
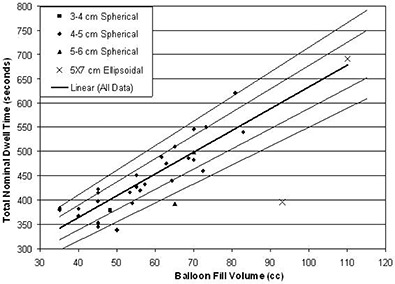
The total nominal dwell time with balloon fill volume is shown for each balloon size for a prescibed dose of 3.4 Gy per fraction to the PTV. A linear regression trend line for all data points $\left(R^{2}=0.81\right)$ is shown (central dark line). The ±7% and ±13% deviation from this linear regression (lighter lines) are shown.

**Figure 3:**
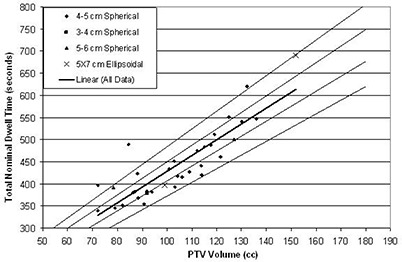
The total nominal dwell time with PTV is shown for each balloon size for a prescibed dose of 3.4 Gy per fraction to the PTV. A linear regression trend line for all data points $\left(R^{2}=0.76\right)$ is shown (central dark line). The ±5% and ±13% deviation from this linear regression (lighter lines) are shown.

The TNDT for each patient is shown for each reported balloon fill and PTV volumes in Figs. 2 and 3, respectively. The balloon size is noted in the legend. A linear regression fit of all data (solid line) is shown in Fig. 3, with $R^{2}=0.81$ and

(1) TNDT=4.48 second/cc×Balloon Fill Volume+185.4 seconds.

Similarly, the trend of TNDT with PTV volume fits linearly to

(2) TNDT=3.55 second/cc×PTV Volume+74.1 seconds.

The thinner lines on each Figure represent the scaled linear fit, which encompasses at least 50% of the data points (inner pair) and at least 90% of the data points (outer pair). For this linear model, 50% of the data points are within 7% and 5% of the linear regression fit for the balloon fill and PTV volume, respectively, and 90% of the data points are within 13% of the fit for both comparisons. It is important to note that these equations for the TNDT correspond only to a prescribed dose of 3.4 Gy per fraction to the PTV.

## IV. DISCUSSION

The Axxent eBx system consists of a miniature electronic X‐ray source, a balloon applicator and a mobile controller. A flexible tungsten‐filled shield, Flexishield (Xoft Inc.), is often placed over the patient during radiation therapy to reduce ambient exposure to the patient and staff. The MammoSite Ir‐192 brachytherapy (IB) system consists of a balloon applicator and a high‐dose rate (HDR) Ir‐192 remote afterloader, which must be used in a shielded radiation vault. Both of these forms of APBI deliver a prescription dose of 3.4 Gy in 10 fractions over five days. The dose rate of the eBx source exceeds that of an HDR Ir‐192 source; however, the eBx source has lower photon energies and a much steeper dose fall‐off with distance. The increased normal tissue sparing as seen in the heart and lung statistics in this study is attributed to the rapid dose fall‐off of the eBx source. The present results are consistent with those reported by Dickler et al.,^(^ ^9^ ^)^ who compared the treatment plans of 15 patients treated with Ir‐192 for localized breast cancer with simulated eBx treatment plans for the same 15 patients. The analyses showed comparable target volume coverage with the two sources; however, the eBx treatment plan had an increased high‐dose region within the target volume, a decreased dose to normal tissue including the ipsilateral lung, and, for patients with tumors on the left side, a decreased dose to the heart. Similar reductions in dose to surrounding normal tissue were also seen with a later comparison of actual Ir‐192 treatment plans and simulated eBx plans for patients with endometrial cancer.^(^ ^14^ ^)^ Similarly, the PTV coverage was found comparable between the two sources, though the high‐dose regions within the PTV were increased and the normal tissue sparing was greater with eBx.^(^ ^14^ ^)^

Although the calculated maximum rib doses were similar between the eBx and Ir‐192 plans, it must be noted that the TG‐43U method does not accurately reflect the absorbed dose‐to‐bone at lower photon energies.^(^ ^12^ ^,^ ^13^ ^)^ The mean energy for the Axxent 50 kVp source ranges from 28–34 keV, similar to the photon energy of I‐125(28keV). The absorbed dose‐to‐bone compared to tissue is considerably higher at these energies, reportedly 4–5 times greater than the absorption in tissue. At least one study has investigated this effect of increased bone dose absorption for therapeutic brachytherapy using I‐125.^(^ ^15^ ^)^ This study reviewed 74 cases where I‐125 seeds were placed almost directly on bone (e.g., pelvis, rib, mandible). The authors in that study found that after one to four years of follow‐up, there was no radiographic evidence of osteoradionecrosis or excessive osseous toxicity. The authors noted that while the dose absorbed by the first 1–2 mm of bone may be very high, there was little dose that penetrated to the underlying marrow or bone layers. In Ir‐192 breast treatments, rib fractures have been reported in 2%–4% of cases. However, no clear correlation between dose and likelihood of fracture has been established.^(^ ^16^ ^)^ In the one‐year follow‐up of eBx APBI treatments, no rib fractures have yet been noted;^(^ ^11^ ^)^ however, longer follow‐up may be required to determine the risk of osteoradionecrosis. Additionally, larger %V150 and %V200s with interstitial brachytherapy have been associated with an increased risk of developing fat necrosis.^(^ ^17^ ^)^ A fat necrosis occurrence rate of 5.5% at one year following treatment with eBx has recently been reported.^(^ ^11^ ^)^ Longer follow‐up is needed to further assess the risk of fat necrosis in patients treated with eBx.

Many factors can influence a final treatment plan including TPS accuracy and precision, acceptable dose volume histogram (DVH) coverage (e.g., 95% or 90%), the centering of the source channel in the implanted balloon, and nonspherical PTVs. It is not expected that a final plan could be generated from only the fill volume parameters. However, as 90% of the total nominal dwell times are within 13% of the linear regression of the balloon fill volume, the linear regression fit could be used as a quality metric to evaluate a plan. All TNDTs were within 13% of either the fill volume trend or the PTV trend. For example, in Fig. 2, the extreme outlier point in the lower right represents a 5×7 ellipsoidal balloon, which was filled to $93\;{\text{cm}}^{3}$, the lower end of the manufacturer's recommended limits $(90–135\;{\text{cm}}^{3}$). However, the 399.0 second TNDT predicted by the 99.$0\;{\text{cm}}^{3}$ PTV for this balloon was within 13% of the expected trend. A potential method for quality assessment of any given plan would be to verify that the TNDT falls within the 13% limits of either, or both, volume‐based metrics as described in Eq. (1) and (2).

Recent advances in brachytherapy modeling which account for suboptimal scattering conditions at skin or lung boundaries will change this dosimetry. For example, skin doses are reportedly overestimated by as much as 10%–15% for Ir‐192.^(^ ^18^ ^)^ A similar scatter effect for skin dose may, or may not, be noticed for eBx. As techniques improve for modeling skin, lung and bone doses, a retrospective review of dosimetric results originally obtained using TG‐43U models will be useful to correlate the clinical results with the improved dosimetry.

## V. CONCLUSIONS

This dosimetric comparison of Ir‐192 and eBx sources demonstrated that both forms of balloon‐based brachytherapy provide comparable dose to the PTV in the delivery of accelerated partial breast irradiation. EBx is associated with increased high‐dose areas within the PTV. The steeper dose fall‐off with the eBx source resulted in appreciably increased normal tissue sparing in the heart and ipsilateral lung. The long‐term effects of increased absorbed rib dose cannot be assessed at this time. A quality assurance metric incorporating the balloon fill and PTV volumes to check the total dwell time generated by the TPS has been proposed.

## ACKNOWLEDGEMENTS

The authors wish to thank all site participants and investigators who supported this research, and the patients and their families for participating in this study. Funding for this study was provided by Xoft, Inc.
